# A Transgenic Mouse Model of Eccentric Left Ventricular Hypertrophy With Preserved Ejection Fraction Exhibits Alterations in the Autophagy-Lysosomal Pathway

**DOI:** 10.3389/fphys.2021.614878

**Published:** 2021-04-22

**Authors:** Kristin Wenzel, Elisabeth Krämer, Birgit Geertz, Lucie Carrier, Stephan B. Felix, Stephanie Könemann, Saskia Schlossarek

**Affiliations:** ^1^Department of Internal Medicine B, University Medicine Greifswald, Greifswald, Germany; ^2^German Centre for Cardiovascular Research (DZHK), partner site Greifswald, Greifswald, Germany; ^3^Institute of Experimental Pharmacology and Toxicology, University Medical Center Hamburg-Eppendorf, Hamburg, Germany; ^4^German Centre for Cardiovascular Research (DZHK), partner site Hamburg/Kiel/Lübeck, Hamburg, Germany

**Keywords:** autophagy-lysosomal pathway, cardiomyopathy, left ventricular hypertrophy, protein degradation, ubiquitin-proteasome system

## Abstract

The ubiquitin-proteasome system (UPS) and the autophagy-lysosomal pathway (ALP) are the main proteolytic systems involved in cellular homeostasis. Since cardiomyocytes, as terminally differentiated cells, lack the ability to share damaged proteins with their daughter cells, they are especially reliant on these protein degradation systems for their proper function. Alterations of the UPS and ALP have been reported in a wide range of cardiac diseases, including cardiomyopathies. In this study, we determined whether the UPS and ALP are altered in a mouse model of eccentric left ventricular (LV) hypertrophy expressing both cyclin T1 and Gαq under the control of the cardiac-specific α-myosin heavy chain promoter (double transgenic; DTG). Compared to wild-type (WT) littermates, DTG mice showed higher end-diastolic (ED) LV wall thicknesses and diameter with preserved ejection fraction (EF). The cardiomyopathic phenotype was further confirmed by an upregulation of the fetal gene program and genes associated with fibrosis as well as a downregulation of genes involved in Ca^2+^ handling. Likewise, higher NT-proBNP levels were detected in DTG mice. Investigation of the UPS showed elevated steady-state levels of (poly)ubiquitinated proteins without alterations of all proteasomal activities in DTG mice. Evaluation of ALP key marker revealed a mixed pattern with higher protein levels of microtubule-associated protein 1 light chain 3 beta (LC3)-I and lysosomal-associated membrane protein-2, lower protein levels of beclin-1 and FYVE and coiled-coil domain-containing protein 1 (FYCO1) and unchanged protein levels of p62/SQSTM1 in DTG mice when compared to WT. At transcriptional level, *a* > 1.2-fold expression was observed for *Erbb2*, *Hdac6*, *Lamp2*, *Nrg1*, and *Sqstm1*, while *a* < 0.8-fold expression was revealed for *Fyco1* in DTG mice. The results related to the ALP suggested overall a repression of the ALP during the initiation process, but an induction of the ALP at the level of autophagosome-lysosome fusion and the delivery of ubiquitinated cargo to the ALP for degradation.

## Introduction

The adult heart undergoes distinct remodeling processes in response to acute or chronic insults, which involve myocardial hypertrophy, ventricular wall thickening and dilatation as well as cardiomyocyte apoptosis and the development of fibrosis (Frey and Olson, 2003; Dorn and Force, 2005). Cardiac myocytes are terminally differentiated and maintain their cellular homeostasis by activation of degradation programs. The two main proteolytic systems are the ubiquitin-proteasome system (UPS) and the autophagy-lysosomal pathway (ALP). The highly selective degradation process by the UPS is ATP-dependent and involves the polyubiquitination of a target protein through a series of enzymatic reactions and the subsequent degradation of this (poly)ubiquitinated protein by the 26S proteasome (Ciechanover, 2007). During the proteolytic process of ALP small portions of the cytoplasm or complete organelles are enclosed by a phagophore to form a double−membrane vesicle, termed autophagosome, which subsequently fuses with a lysosome to form an autolysosome, in which lysosomal proteases degrade the autophagosomal content (Mizushima et al., 2008). Several lines of evidence indicate that alterations of the UPS and the ALP may be involved in cardiac diseases, such as dilated and hypertrophic cardiomyopathies (Mearini et al., 2008; Zheng and Wang, 2010; Day, 2013; Zech et al., 2020). A marked accumulation of (poly)ubiquitinated proteins has been reported as a common feature of cardiac disorders (Weekes et al., 2003; Birks et al., 2008; Predmore et al., 2010), whereas the proteasomal activities have been shown to be higher or lower depending on the status of the cardiac disease (Depre et al., 2006; Tsukamoto et al., 2006; Birks et al., 2008; Predmore et al., 2010; Schlossarek et al., 2012; Thottakara et al., 2015). Likewise, an altered autophagic flux and dysregulated expression of ALP key marker have been observed in a wide range of cardiac diseases such as desmin-related cardiomyopathy (Maloyan et al., 2010; Pattison et al., 2011; Bhuiyan et al., 2013), dilated cardiomyopathy (Choi et al., 2012; Ramos et al., 2012) and hypertrophic cardiomyopathy with reduced ejection fraction (EF; Schlossarek et al., 2012; Singh et al., 2017). The role of the ALP in the development of cardiac hypertrophy and its progression to heart failure is being discussed controversially (Zhu et al., 2007; Rothermel and Hill, 2008). Currently, a basal ALP activity is thought to be important to maintain normal cardiac function, whereas a decrease or an increase in ALP activity could mediate the adaption of the heart during stress conditions (Lavandero et al., 2013).

Traditionally, the UPS and ALP have been considered to act separately, but recent data suggest that they functionally cooperate with each other to maintain cellular homeostasis (Wang et al., 2008; Korolchuk et al., 2010; Lamark and Johansen, 2010; Singh et al., 2020). In the present study, we therefore aimed to investigate alterations of both the UPS and the ALP in a mouse model of eccentric left ventricular (LV) hypertrophy with preserved EF expressing both cyclin T1 and Gαq under the control of the cardiac-specific α-myosin heavy chain promoter.

## Materials and Methods

### Ethics Statement

All experimental procedures were conducted in compliance with the national guidelines of the German animal protective law for the use of laboratory animals. All animal procedures conform to the guidelines of Directive 2010/63/EU of the European Parliament on the protection of animals used for scientific purposes. The protocols used were approved by the local animal care committee (Landesamt für Landwirtschaft, Lebensmittelsicherheit und Fischerei Mecklenburg-Vorpommern; LALLF-MV, 7221.3-1-061/15).

### Experimental Animals

Double transgenic (DTG) α-myosin heavy chain (αMHC)-cyclin T1 × αMHC-Gαq mice on a Friend leukaemia virus strain (FVB) background of either sex were obtained by crossbreeding heterogeneous αMHC-cyclin T1 mice, kindly provided by Prof. Michael D. Schneider (Imperial College London, United Kingdom), with αMHC-Gαq overexpressing mice, courtesy of Prof. Gerald W. Dorn 2nd (Washington University, St. Louis, United States). The heart-specific activation of Cdk9 via forced expression of cyclin T1 induces LV hypertrophy by phosphorylation of the RNA polymerase II (Sano et al., 2004). Gαq overexpression further enhances the transcription elongation, thereby serving as an additional hypertrophic stimulus (D’Angelo et al., 1997). Gαq overexpression results in cardiac hypertrophy, defined as a conserved program of fetal gene expression, increased heart weight, and increased cardiomyocyte size. Thus, the DTG mice show a global increase in RNA synthesis leading to myocyte enlargement and hypertrophic growth and further development of fibrosis as well as apoptosis.

Wild-type (WT) littermates were used as healthy controls and both genotypes were investigated at the age of 6–8 weeks.

### Study Design and Tissue Processing

Cardiac function of 6–8-weeks-old mice was assessed via magnetic resonance imaging (MRI). Afterward, the mice were sacrificed while still being under isoflurane anesthesia. Hearts were harvested and approximately 500 μL whole blood was collected. Organs were washed in PBS and subsequently frozen at −80°C. Blood was transferred to an Eppendorf tube containing 0.3 μM EDTA (10 μL/200 μL blood) and centrifuged at 300 g for 15 min at room temperature. Plasma was transferred to a new Eppendorf tube and stored at −20°C.

### Magnetic Resonance Imaging Measurement

Anesthesia was induced using 4% isoflurane in 100% oxygen and maintained with 2–3% isoflurane in 100% oxygen at a flow rate of 1.0 L/min using a face mask during the procedure. The depth of anesthesia was monitored using a pressure sensor for respiration. Breathing rates were maintained at 30–40 breaths per minute. Body temperature was continuously measured via a rectal thermal probe and kept at 35°C using a flowing-water heating blanket. Heart function was measured using a 7.1 Tesla MR system (ClinScan 70/30 30 Bruker, Ettlingen, Germany) with 290 mT/m gradients field strength. For signal excitation a head coil was used, while signal reception was realized using a four-channel rat brain array. After three localizer sequences and a fast single shot fast low angle shot (FLASH) single shot axial sequence, as 2 chamber view (2 CHV) and as 4 chamber view (4 CHV) were acquired. In addition, prospective electrocardiogram triggered and respiration gated CINE sequences were generated in 2 CHV [repetition time (TR): 5.8 ms, echo time (TE) 2.37 ms, field-of-view (FoV): 35 × 35 mm, FoV Phase: 100%, flip angle: 20°, slice thickness 0.7 mm] and 4 CHV (TR: 5.8 ms, TE: 2.37 ms, FoV: 35 × 35 mm, FoV Phase: 100%, flip angle: 20°, slice thickness 0.7 mm) were obtained. Afterward, five short axis CINE (flash) sequences of the left ventricle (TR: 5.7 ms, TE: 2.25 ms, FoV: 35 × 35 mm, FoV Phase: 100%, flip angle: 25°, slice thickness: 1.0 mm) were acquired. The results were analyzed using the software Segment, version 1.9 R3510 (Medviso and Osirix, version 5.8.2 32-bit, Lund, Sweden). The endocardium and epicardium were delineated in all five short axes in both end-diastole and end-systole, which the software used to calculate the specific area in μm^2^. Papillary muscles were included in the delineation of the endocardium. Followed by a conversion in a specific volume (in μm^3^), utilizing a slice thickness of 1 mm, left ventricular end-diastolic volume (LVEDV) was acquired by using the volume from each slice. The end-diastole was determined as being the phase with the largest area of the LV cavity and the end-systole being the one with the smallest area in each slice. The EF was calculated with the help of LVEDV and left ventricular end-systolic volume (LVESV) (EF = [(LVEDV − LVESV)/LVEDV] × 100). Fractional shortening (FS) was calculated using the left mid-ventricular diameter (FS = [(LVEDD − LVESD)/LVEDD] × 100). The LV mass was computed by multiplying the LV volume by the specific density of a healthy (assuming the lack of infarcted regions in the heart) myocardium of 1.05 g/ml.

### ELISA

Mouse plasma N-terminal pro-brain natriuretic peptide (NT-proBNP) concentrations were measured using ELISA kits (SEA485Mu, Cloud Clone Corp., Houston, TX, United States) according to the instructions of the manufacturer.

### RNA Extraction and Expression Analysis With the NanoString nCounter® Elements

Total RNA was extracted from powdered whole heart tissue samples using the SV Total RNA isolation kit (Promega, Madison, WI, United States) according to the manufacturer‘s instructions.

For gene expression analysis, a customized NanoString’s nCounter Elements TagSet panel was used. About 50 ng of each sample were hybridized to the target-specific capture and reporter probes at 67°C overnight (16 h) according to manufacturer’s instructions. Samples were cooled down to 4°C, supplemented with 15 μl H_2_O, and loaded into the NanoString cartridge. Afterward, the nCounter Gene Expression Assay was started immediately. Raw data were analyzed with the nCounter Sprint Profiler. Transcript levels were determined with the nSolver Data Analysis Software including background subtraction using negative controls and normalization to six housekeeping genes (*Abcf1*, *Actb*, *Cltc*, *Gapdh*, *Pgk1*, and *Tubb5*). Data represent the mean of normalized counts and are expressed as fold change over WT.

### Analysis of Protein Degradation Pathways UPS and ALP

#### Sample Preparation

Tissue samples were powdered, and protein extraction was performed in two steps. First, the organ powder (about 30 mg) was dissolved in 150 μl water with a protease inhibitor cocktail (complete miniTM, Roche Diagnostics, Rotkreuz, Switzerland). After three freeze-thaw-cycles the tissue was homogenized by using Tissue Lyser (2 × 30 s at 20 Hz) and centrifuged at 4°C, full speed for 30 min in a table-top centrifuge. The supernatant was kept as the cytosolic fraction. Secondly, the pellet of the first step was homogenized in 240 μl SDS-buffer (3% SDS, 30 mM Tris-base, pH 8.8, 5 mM EDTA, 30 mM NaF, 10% glycerol and 1 mM DTT) and centrifuged at room temperature, full speed for 10 min in a table-top centrifuge. The supernatant was kept as the membrane-enriched fraction.

#### Western Blot

Proteins of the membrane-enriched fraction were loaded on acrylamide/bisacrylamide (29:1) gels and electrotransferred to nitrocellulose membranes, except for LC3 analysis, for which proteins were electrotransferred to polyvinylidene fluoride membranes. Antibodies against the following proteins were used for western blot analysis: Beclin-1 (Cell Signaling Technology, 3738), FYCO1 (Novus Biologicals, NBP1-47266), LAMP-2 (Abcam, ab13524), LC3 (Cell Signaling Technology, 2775), p62 (Sigma Aldrich, P0067), ribosomal protein S6 (Cell Signaling Technology, 2217), and ubiquitinated proteins (Enzo Life Sciences, BML-PW8810). Signals were revealed with the Clarity Western ECL substrate (Bio-Rad) and acquired with the ChemiDoc Touch Imaging System (Bio-Rad). Signals were quantified with the Image Lab Software (Bio-Rad).

### Determination of the 20S and 26S Proteasomal Activities

The activities of the 20S and 26S proteasome were assessed in the cytosolic protein fraction. For determination of the activity, 30 μg of protein were diluted in incubation buffer (20 mM HEPES, 0.5 mM EDTA, 5 mM MgCl_2_, 1 mM DTT) to a final volume of 50 μl. Samples were pre-incubated in this buffer for 2 h at 4°C. Following pre-incubation, the synthetic fluorogenic substrates Suc-LLVY-AMC (Enzo Life Sciences, BML-P802), Z-LLE-AMC (Enzo Life Sciences, BML-ZW9345) and Ac-RLR-AMC (Enzo Life Sciences, BML-AW9785) were added to the samples at a final concentration of 60 μM, 45 μM and 40 μM in the presence and absence of 28, 14, and 14 μM ATP for chymotrypsin-like, caspase-like and trypsin-like activity, respectively. After incubation in the dark for 1 h at 37°C, the fluorescence of the released AMC reporters was measured using the TECAN Safire2 microplate reader at an excitation wavelength of 380 nm and an emission wavelength of 460 nm. Each sample was measured in duplicate. The mean of the blank (incubation buffer only) was subtracted from the mean of each sample duplicate.

### Data Analysis

Data are presented as mean ± SEM. Statistical analyses were performed using the unpaired Student’s *t*-test. All analyses were realized using GraphPad Prism 8. A value of *p* < 0.05 was considered statistically significant.

## Results

### DTG Mice Exhibit Eccentric LV Hypertrophy With a Preserved Systolic Function

**FIGURE 1:**
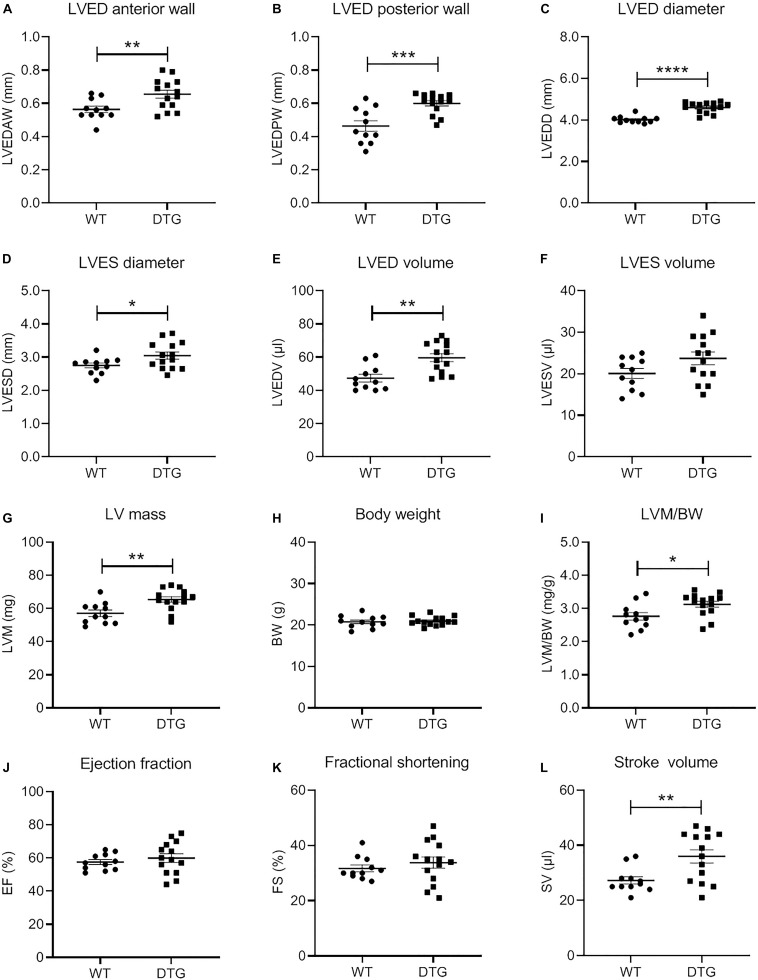
Evaluation of morphological and functional parameters of the heart of DTG and WT mice. Scatter plots show the difference in **(A)** left ventricular (LV) end-diastolic (ED) anterior wall thickness (LVEDAW), **(B)** LVED posterior wall thickness (LVEDPW), **(C)** LVED diameter (LVEDD), **(D)** LV end-systolic (ES) diameter (LVESD), **(E)** LVED volume (LVEDV), **(F)** LVES volume (LVESV), **(G)** LV mass (LVM), **(H)** body weight (BW), **(I)** LVM/BW, **(J)** ejection fraction (EF), **(K)** fractional shortening (FS), and **(L)** stroke volume (SV) of DTG and WT mice. Data are presented as mean ± SEM with *p < 0.05, **p < 0.01, ***p < 0.001, and ****p < 0.0001 vs. WT, unpaired Student’s *t*-test. *N* = 11–14.

Representative MRI images of WT and DTG hearts are shown in Supplementary Figure 1. MRI measurements of the DTG mice showed significantly higher LVED anterior and posterior wall thicknesses (Figures 1A,B), LVED and LVES diameters (Figures 1C,D), and LVED volumes (Figure 1E) than WT mice, whereas LVES did not differ (Figure 1F). In line with this, the LV mass (Figure 1G), but not the body weight (Figure 1H), and LV mass/body weight ratio (Figure 1I) were higher in DTG than WT mice. In contrast, no difference in EF and FS was observed between the two groups (Figures 1J,K). The stroke volume was higher in DTG than WT mice (Figure 1L). Thus, DTG mice showed eccentric LV hypertrophy with preserved systolic function.

### DTG Mice Show Typical Gene Expression Changes Associated With Pathological Cardiac Hypertrophy

To further examine the disease state of the DTG mice, the expression of several genes related to cardiac hypertrophy, fetal gene program, fibrosis, apoptosis and Ca^2+^ handling was evaluated by using the NanoString’s nCounter Elements technology (Table 1). While expression levels of genes related to hypertrophy (*Fhl1* and *Rcan1*) were not significantly higher, the expression of *Fhl2*, which has been shown to be down-regulated during hypertrophy, was markedly lower in DTG mice. The expression level of genes associated with the reactivation of the fetal gene program (*Nppa*, *Nppb*, and *Myh7*) was significantly higher in DTG than WT mice. Genes related to fibrosis (*Col1a1*, *p* < 0.001 and *Col3a1*, *p* = 0.06) and genes specific for fibroblasts (*Ctgf*, *S100a4* and *Postn*) were up-regulated in DTG mice. Furthermore, the expression of *Bcl2*, a key regulator of apoptosis, and *Vwf*, a marker of endothelial dysfunction, was higher in DTG mice. In contrast, genes involved in Ca^2+^ handling (*Atp2a2*, *Pln*, and *Ryr2*) were downregulated in DTG mice.

**TABLE 1:** Gene expression analysis in DTG and WT mice.

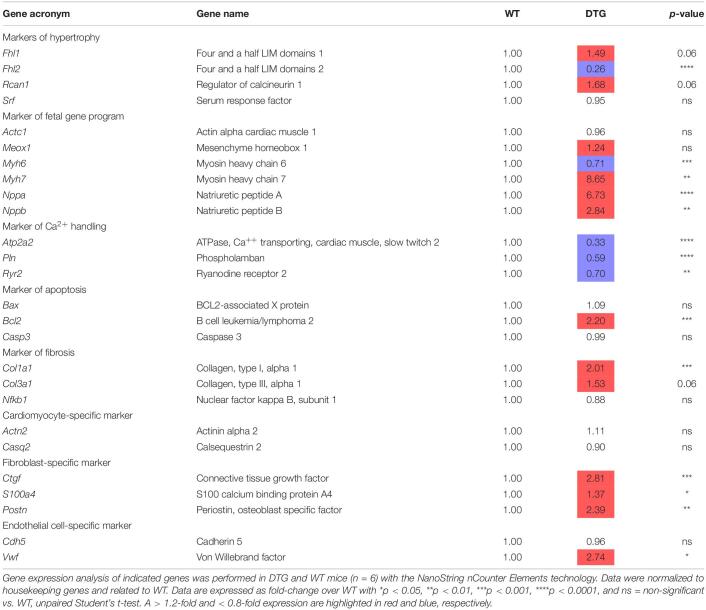

Gene expression analysis of indicated genes was performed in DTG and WT mice (*n* = 6) with the NanoString nCounter Elements technology. Data were normalized to housekeeping genes and related to WT. Data are expressed as fold-change over WT with *p < 0.05, **p < 0.01, ***p < 0.001, ****p < 0.0001, and ns = non-significant vs. WT, unpaired Student’s *t*-test. *A* > 1.2-fold and < 0.8-fold expression are highlighted in red and blue, respectively.

### DTG Mice Express Higher Level of NT-proBNP

**FIGURE 2:**
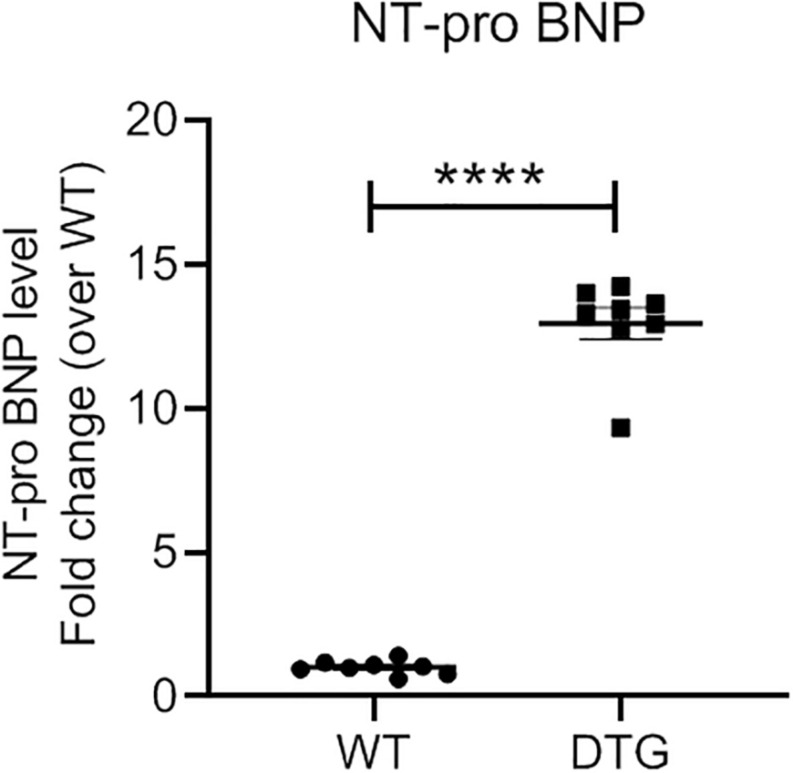
Plasma NT-proBNP level in DTG and WT mice. Data are presented as mean ± SEM with ****p < 0.0001 vs. WT, unpaired Student’s *t*-test. *N* = 8.

NT-proBNP is used in clinical routine as a prognostic biomarker for LV hypertrophy and heart failure. The NT-proBNP level was ∼13-fold higher in DTG than in WT mice (Figure 2). This is in line with the results of the gene expression analysis for the natriuretic peptides *Nppa* and *Nppb* which were higher expressed in DTG mice than in healthy littermates.

### DTG Mice Display Accumulation of (Poly)ubiquitinated Proteins Without Alteration of Proteasomal Activities

**FIGURE 3:**
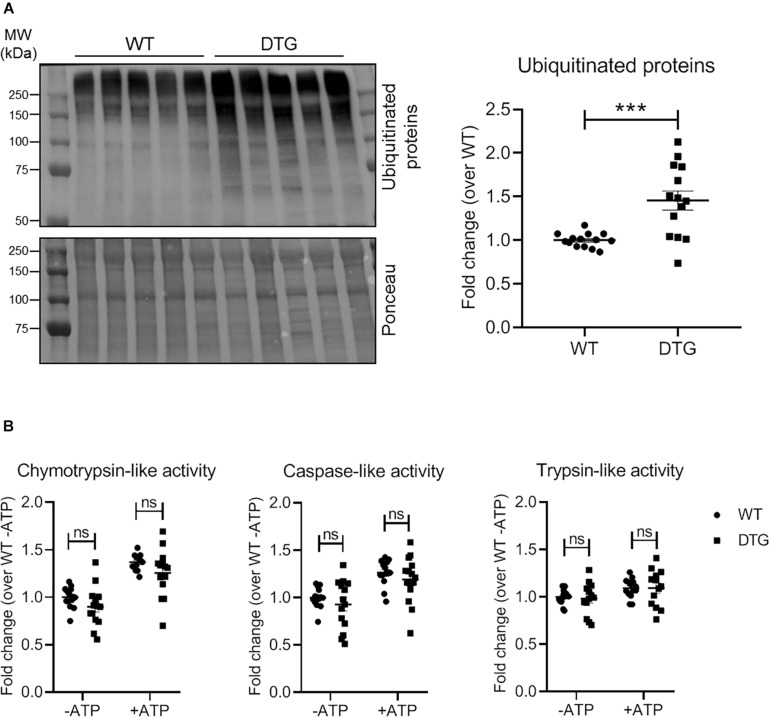
Steady-state levels of ubiquitinated proteins and proteasomal activities in DTG and WT mice. **(A)** Representative Western blot and quantification of the steady-state levels of ubiquitinated proteins normalized to Ponceau and related to WT. **(B)** 20S (ATP-independent) and 26S (ATP-dependent) chymotrypsin-like, caspase-like and trypsin-like activities were measured by using specific fluorogenic substrates. Data are presented as mean ± SEM with ***p < 0.001 vs. WT and ns = non-significant, unpaired Student’s *t*-test. *N* = 13–14.

Since accumulation of (poly)ubiquitinated proteins and altered proteasomal activities have been reported in cardiac diseases, we determined the steady-state levels of (poly)ubiquitinated proteins by Western blot and measured the 20S (ATP-independent) and 26S (ATP-dependent) proteasomal activities by using synthetic fluorogenic substrates in heart tissue of DTG and WT mice. The steady-state level of polyubiquitinated proteins was 1.5-fold higher in DTG than in WT mice (Figure 3A). Additionally, cardiac sections have been stained with a monoclonal antibody directed against ubiquitinated proteins. In agreement with the Western blot analysis, the number of dots of (poly)ubiquitinated proteins was significantly higher in DTG than in WT mice (Supplementary Figure 2 and Supplementary Table 1). In contrast, the 20S and 26S chymotrypsin-like, caspase-like and trypsin-like activities did not differ between DTG and WT mice (Figure 3B).

### DTG Mice Show an Altered ALP

**FIGURE 4:**
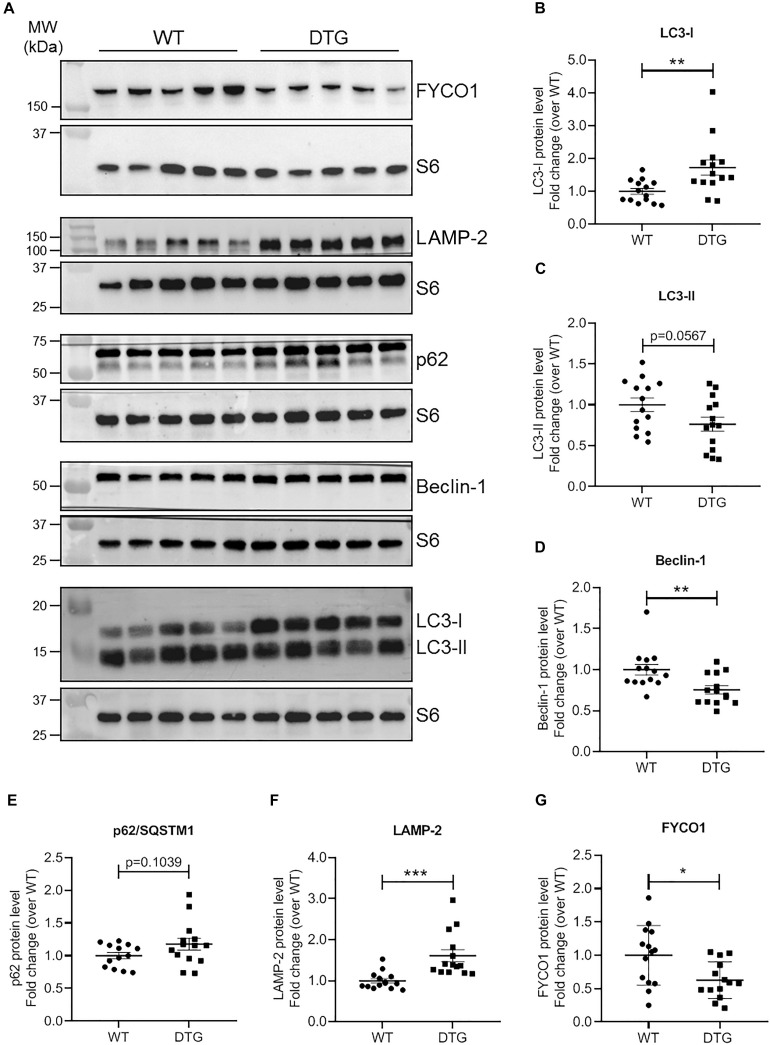
ALP marker proteins in DTG and WT mice. **(A)** Representative Western blots of indicated proteins. S6 ribosomal protein was used as loading control. Quantification of **(B)** LC3-I, **(C)** LC3-II, **(D)** Beclin-1, **(E)** p62/SQSTM1, **(F)** LAMP-2, and **(G)** FYCO1 protein levels normalized to S6 ribosomal protein and related to WT. Data are presented as mean ± SEM with *p < 0.05, **p < 0.01 and ***p < 0.001 vs. WT, unpaired Student’s *t*-test. *N* = 13–14.

To identify the regulation of autophagic processes in the disease pattern of cardiac hypertrophy, several markers involved in the process of the ALP were determined using the NanoString’s nCounter Elements technology (Table 2) and Western blot analysis (Figure 4).

**TABLE 2:** Autophagy-lysosomal pathway gene expression analysis in DTG and WT mice.

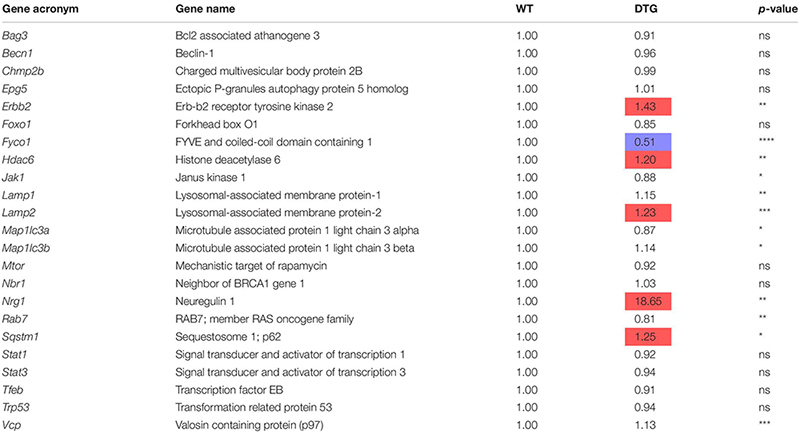

Gene expression analysis of indicated genes was performed in DTG and WT mice (*n* = 6) with the NanoString nCounter Elements technology. Data were normalized to housekeeping genes and related to WT. Data are expressed as fold-change over WT with *p < 0.05, **p < 0.01, ***p < 0.001, ****p < 0.0001, and ns = non-significant vs. WT, unpaired Student’s *t*-test. A ≥ 1.2-fold and < 0.8-fold expression are highlighted in red and blue, respectively.

Microtubule-associated protein 1 light chain 3 beta (hereafter referred to as LC3) is a central protein in the autophagy pathway and exists in a soluble form termed LC3-I and a lipidated form referred to as LC3-II. LC3-II is incorporated into the autophagosomal membrane and is important for autophagosome formation and maturation, shuttling and fusion with the lysosomes (Zech et al., 2020). Whereas *Map1lc3b* transcript and LC3-I protein levels were higher in DTG mice, LC3-II protein levels tended to be lower in DTG than WT mice (Table 2 and Figures 4A–C). Next, we evaluated beclin-1, which is involved in the phagophore structure initiation, an early step of autophagy (Zech et al., 2020). Beclin-1 protein levels were lower in DTG than WT mice, while beclin-1 mRNA levels did not differ between the groups (Figures 4A,D and Table 2). Furthermore, the protein levels of p62/SQSTM1, which acts as a shuttle protein that binds ubiquitinated proteins and LC3-II and directs ALP-mediated degradation of ubiquitinated proteins (Pankiv et al., 2007), did not differ between DTG and WT mice, whereas the expression of *Sqstm1* was induced on transcriptional level in DTG mice (Figures 4A,E and Table 2). The expression of the lysosomal-associated membrane protein-2 (LAMP-2) was higher on both mRNA and protein levels in DTG than WT mice (Table 2 and Figures 4A,F). In contrast, the expression of FYVE and coiled-coil domain-containing protein 1 (FYCO1), which forms an adaptor protein complex with LC3 and Rab7 (Pankiv et al., 2010), was lower on both mRNA and protein levels in DTG than WT mice (Table 2 and Figures 4A,G). A > 1.2-fold expression was further observed for *Erbb2*, *Hdac6* and *Nrg1* at transcriptional level in DTG mice (Table 2).

## Discussion

In the present study we investigated alterations of the two major proteolytic systems namely UPS and ALP in a mouse model of eccentric LV hypertrophy with preserved EF. The major findings of the present study are: (1) accumulation of (poly)ubiquitinated proteins, (2) no alteration of the 20S and 26S proteasomal activities, (3) higher protein levels of LC3-I and LAMP-2, (4) lower protein levels of beclin-1 and FYCO1, (5) > 1.2-fold expression for *Erbb2*, *Hdac6*, *Lamp2*, *Nrg1*, and *Sqstm1*, and (6) < 0.8-fold expression for *Fyco1* at transcriptional level in DTG mice. These findings indicate an altered autophagic clearance of damaged and/or dysfunctional proteins in a mouse model of eccentric LV hypertrophy.

In recent years, the UPS was found to play a fundamental role in several biological processes including cell proliferation, adaption to stress and cell death (Rock et al., 1994; King et al., 1996; Breitschopf et al., 2000). Various studies found that activation or impairment of the UPS is associated with different cardiac diseases (Mearini et al., 2008; Su and Wang, 2010; Schlossarek and Carrier, 2011; Day, 2013). An accumulation of (poly)ubiquitinated proteins was documented in failing human hearts (Weekes et al., 2003; Birks et al., 2008; Predmore et al., 2010). In our transgenic mouse model showing eccentric LV hypertrophy with preserved EF the classical signature of a hypertrophic response including the up-regulation of the genes encoding BNP (*Nppb*), ANP (*Nppa*) and α-myosin heavy chain (*Myh7*) was detected together with higher levels of (poly)ubiquitinated proteins and without alterations of proteasomal activities.

The UPS and ALP have long been considered to act separately, but recent data suggest that they work as a consortium (Wang et al., 2008; Korolchuk et al., 2010; Lamark and Johansen, 2010; Singh et al., 2020). Perturbations in the degradation process of either pathway have been reported to affect the activity of the other one (Korolchuk et al., 2010; Pan et al., 2020). For instance, impairment of the UPS has been shown to trigger autophagy (Iwata et al., 2005; Pandey et al., 2007), suggesting autophagic upregulation as a compensatory mechanism. Evaluation of the ALP key marker LC3 showed that LC3-II protein levels tended to be lower in DTG than WT mice, whereas *Map1lc3b* transcript and LC3-I protein levels were higher in DTG mice. Lower LC3-II protein level could indicate an alteration in the lipidation process from LC3-I to LC3-II (Kabeya et al., 2000). On the other hand, LC3-II is a substrate of the ALP as well and lower levels of LC3-II protein could as well indicate a rapid degradation via the ALP. The elevated *Map1lc3b* transcript and LC3-I protein levels support more the second scenario suggesting an activated or at least proper working ALP in the DTG mice. Further support for an activated ALP is the increased expression of LAMP-2 on both mRNA and protein levels in the DTG mice, indicating an intact autophagosome-lysosome fusion and an enhanced lysosomal activity. Autophagosome-lysosome fusion occurs in the vicinity of the centrosome. To get to the perinuclear region, autophagic vesicles have to move along the microtubules and this interaction with the microtubules is mediated by motor proteins (Mackeh et al., 2013). So-called plus-end-directed motor proteins transport autophagic vesicles toward the cellular periphery and minus-end-directed motor proteins mediate the transport to the perinuclear region (Hirokawa et al., 2009). The balance between active plus-end- and minus-end-directed motor proteins on the surface of autophagic vesicles determines the directionality of their intracellular movement (Mackeh et al., 2013). FYCO1 forms an adaptor protein complex with LC3 and Rab7 and promotes the plus-end-directed transport of autophagic vesicles (Pankiv et al., 2010). We observed markedly reduced levels of FYCO1 on both transcript and protein levels in DTG mice, suggesting that the directionality of the intracellular movements of autophagic vesicles is maybe shifted toward the perinuclear region.

Protein levels of p62/SQSTM1 did not differ between DTG and WT mice. This protein serves as a linker between ubiquitinated proteins and LC3-II thereby directing ALP-mediated degradation of ubiquitinated proteins (Pankiv et al., 2007) and is as well degraded by the ALP (Bjorkoy et al., 2005). The induced expression of *Sqstm1* at transcriptional level may indicate an increased demand for p62/SQSTM1. Unaltered p62/SQSTM1 protein level in spite of elevated transcript level could indicate a proper or even induced working ALP degrading its substrate. Another protein serving as a linker between ubiquitinated cargo and the ALP is the histone deacetylase 6 (HDAC6). HDAC6 binds ubiquitin and interacts directly with dynein motors, thereby ensuring efficient delivery of substrates to the ALP for degradation (Kawaguchi et al., 2003). A protective effect of compensatory upregulation of autophagy during proteasomal inhibition has been proposed to be dependent on HDAC6 (Iwata et al., 2005; Pandey et al., 2007). We did not determine the protein levels, but found as well elevated *Hdac6* transcript levels in the DTG mice suggesting similar to p62/SQSTM1 an induced expression to deal with the higher amounts of ubiquitinated proteins. Interestingly, beclin-1 protein levels were lower in DTG than WT mice, while *Becn1* mRNA levels did not differ between the groups. Beclin-1 is involved in the phagophore structure initiation (Zech et al., 2020), and lower protein level suggest, in contrast to our other data, rather a repression of the ALP, at least during the initiation process. Bcl-2 (B-cell leukemia/lymphoma 2), which was significantly up-regulated at the transcriptional level in the DTG mice, is directly linked to the autophagy process. Bcl-2 interacts with beclin-1 by inhibiting the formation of the beclin-1/Vsp34 PI3K complex and therefore the beclin-1-dependent autophagy (Pattingre et al., 2005). Pattingre et al. (2005) proposed that this anti-autophagic action of Bcl-2 may help to maintain autophagy at an optimal level for cell survival rather than cell death. In addition, Bcl-2 partly mediates the anti-autophagic effects of neuregulin-1 (Nrg-1) (An et al., 2013). Nrg-1 and its corresponding receptor ErbB2 (Erb-b2 receptor tyrosine kinase 2) were significantly up-regulated at the transcriptional level in the hearts of the DTG mice. Nrg-1 induces a number of cellular responses, such as regulation of cell proliferation and differentiation and plays a critical role in cardiovascular development and maintenance of heart function by promoting cardiac myocyte survival and maintenance of Ca^2+^ homeostasis (De Keulenaer et al., 2019). Although there was a marked increase in *Nrg1* expression in the DTG mice, almost all genes involved in Ca^2+^ handling were significantly down-regulated. Nevertheless, the increase in *Nrg1* and its receptor *Erbb2* suggests an induction of survival, structural and functional maintenance and anti-autophagic processes, probably similar to Bcl-2 to maintain autophagy at an optimal level.

Many studies have shown that activation of G-protein-coupled receptors can regulate autophagy (Wauson et al., 2014). Liu et al. (2017) investigated the ALP in a transgenic mouse model of cardiac-specific inducible Gαq expressing a constitutively active GαqQ209L fused to a modified hormone-binding domain of the estrogen receptor (Liu et al., 2017). In contrast to the DTG mice, which exhibit a preserved ejection function, the transgenic GαqQ209L mice showed a reduced FS after 7 days of treatment with tamoxifen. At this time point, Liu et al. reported an activated autophagy in the GαqQ209L mice including elevated protein levels of beclin-1, p62, LC3-II, LAMP-1, and LAMP-2 (Liu et al., 2017).

In conclusion, evaluation of the ALP in the DTG αMHC-cyclin T1 × αMHC-Gαq mice revealed a mixed pattern in that the results suggested a repression of the ALP during the initiation process, but an induction of the ALP at the level of autophagosome-lysosome fusion and the delivery of ubiquitinated cargo to the ALP for degradation.

## Data Availability Statement

The original contributions presented in the study are included in the article/Supplementary Material, further inquiries can be directed to the corresponding author.

## Ethics Statement

The animal study was reviewed and approved by the Landesamt für Landwirtschaft, Lebensmittelsicherheit und Fischerei Mecklenburg-Vorpommern.

## Author Contributions

KW contributed to the conception and design of the study, management of the mouse cohorts, execution of experiments, analysis and interpretation of data, figure preparation, and drafting of the manuscript. EK and BG contributed to the execution of experiments. LC and SK contributed to the conception and design of the study, analysis and interpretation of data, and drafting of the manuscript. SF contributed to the conception and design of the study and drafting of the manuscript. SS contributed to the conception and design of the study, execution of experiments, analysis and interpretation of data, figure preparation, and drafting of the manuscript. All authors critically discussed the results, and reviewed and approved the manuscript before submission.

## Conflict of Interest

The authors declare that the research was conducted in the absence of any commercial or financial relationships that could be construed as a potential conflict of interest.

## References

[B1] An T. Huang Y. Zhou Q. Wei B. Q. Zhang R. C. Yin S. J. (2013). Neuregulin-1 attenuates doxorubicin-induced autophagy in neonatal rat cardiomyocytes. *J. Cardiovasc. Pharmacol.* 62 130–137. 10.1097/FJC.0b013e318291c094 23519142

[B2] Bhuiyan M. S. Pattison J. S. Osinska H. James J. Gulick J. Mclendon P. M. (2013). Enhanced autophagy ameliorates cardiac proteinopathy. *J. Clin. Invest.* 123 5284–5297. 10.1172/JCI70877 24177425 PMC3859422

[B3] Birks E. J. Latif N. Enesa K. Folkvang T. Luong Le A. Sarathchandra P. (2008). Elevated p53 expression is associated with dysregulation of the ubiquitin-proteasome system in dilated cardiomyopathy. *Cardiovasc. Res.* 79 472–480. 10.1093/cvr/cvn083 18375498

[B4] Bjorkoy G. Lamark T. Brech A. Outzen H. Perander M. Overvatn A. (2005). p62/SQSTM1 forms protein aggregates degraded by autophagy and has a protective effect on huntingtin-induced cell death. *J. Cell Biol.* 171 603–614. 10.1083/jcb.200507002 16286508 PMC2171557

[B5] Breitschopf K. Zeiher A. M. Dimmeler S. (2000). Ubiquitin-mediated degradation of the proapoptotic active form of bid. A functional consequence on apoptosis induction. *J. Biol. Chem.* 275 21648–21652. 10.1074/jbc.M001083200 10801801

[B6] Choi J. C. Muchir A. Wu W. Iwata S. Homma S. Morrow J. P. (2012). Temsirolimus activates autophagy and ameliorates cardiomyopathy caused by lamin A/C gene mutation. *Sci. Transl. Med.* 4:144ra102. 10.1126/scitranslmed.3003875 22837537 PMC3700376

[B7] Ciechanover A. (2007). Intracellular protein degradation from a vague idea through the lysosome and the ubiquitin-proteasome system and on to human diseases and drug targeting: nobel Lecture, December 8, 2004. *Ann. N. Y. Acad. Sci.* 1116 1–28. 10.1196/annals.1402.078 18083918

[B8] D’Angelo D. D. Sakata Y. Lorenz J. N. Boivin G. P. Walsh R. A. Liggett S. B. (1997). Transgenic Galphaq overexpression induces cardiac contractile failure in mice. *Proc. Natl. Acad. Sci. U. S. A.* 94 8121–8126. 10.1073/pnas.94.15.8121 9223325 PMC21567

[B9] Day S. M. (2013). The ubiquitin proteasome system in human cardiomyopathies and heart failure. *Am. J. Physiol. Heart Circ. Physiol.* 304 H1283–93. 10.1152/ajpheart.00249.2012 23479263 PMC4073948

[B10] De Keulenaer G. W. Feyen E. Dugaucquier L. Shakeri H. Shchendrygina A. Belenkov Y. N. (2019). Mechanisms of the Multitasking Endothelial Protein NRG-1 as a Compensatory Factor During Chronic Heart Failure. *Circ. Heart Fail.* 12:e006288. 10.1161/CIRCHEARTFAILURE.119.006288 31607147

[B11] Depre C. Wang Q. Yan L. Hedhli N. Peter P. Chen L. (2006). Activation of the cardiac proteasome during pressure overload promotes ventricular hypertrophy. *Circulation* 114 1821–1828. 17043166 10.1161/CIRCULATIONAHA.106.637827

[B12] Dorn G. W. II. Force T. (2005). Protein kinase cascades in the regulation of cardiac hypertrophy. *J. Clin. Invest.* 115 527–537. 10.1172/JCI24178 15765134 PMC1052008

[B13] Frey N. Olson E. N. (2003). Cardiac hypertrophy: the good, the bad, and the ugly. *Annu. Rev. Physiol.* 65 45–79. 10.1146/annurev.physiol.65.092101.142243 12524460

[B14] Hirokawa N. Noda Y. Tanaka Y. Niwa S. (2009). Kinesin superfamily motor proteins and intracellular transport. *Nat. Rev. Mol. Cell Biol.* 10 682–696. 10.1038/nrm2774 19773780

[B15] Iwata A. Riley B. E. Johnston J. A. Kopito R. R. (2005). HDAC6 and microtubules are required for autophagic degradation of aggregated huntingtin. *J. Biol. Chem.* 280 40282–40292. 10.1074/jbc.M508786200 16192271

[B16] Kabeya Y. Mizushima N. Ueno T. Yamamoto A. Kirisako T. Noda T. (2000). LC3, a mammalian homologue of yeast Apg8p, is localized in autophagosome membranes after processing. *EMBO J.* 19 5720–5728. 10.1093/emboj/19.21.5720 11060023 PMC305793

[B17] Kawaguchi Y. Kovacs J. J. Mclaurin A. Vance J. M. Ito A. Yao T. P. (2003). The deacetylase HDAC6 regulates aggresome formation and cell viability in response to misfolded protein stress. *Cell* 115 727–738. 10.1016/S0092-8674(03)00939-5 14675537

[B18] King R. W. Deshaies R. J. Peters J. M. Kirschner M. W. (1996). How proteolysis drives the cell cycle. *Science* 274 1652–1659. 8939846 10.1126/science.274.5293.1652

[B19] Korolchuk V. I. Menzies F. M. Rubinsztein D. C. (2010). Mechanisms of cross-talk between the ubiquitin-proteasome and autophagy-lysosome systems. *FEBS Lett.* 584 1393–1398. 10.1016/j.febslet.2009.12.047 20040365

[B20] Lamark T. Johansen T. (2010). Autophagy: links with the proteasome. *Curr. Opin. Cell Biol.* 22 192–198. 10.1016/j.ceb.2009.11.002 19962293

[B21] Lavandero S. Troncoso R. Rothermel B. A. Martinet W. Sadoshima J. Hill J. A. (2013). Cardiovascular autophagy Concepts, controversies, and perspectives. *Autophagy* 9 1455–1466. 10.4161/auto.25969 23959233

[B22] Liu S. Jiang Y. P. Ballou L. M. Zong W. X. Lin R. Z. (2017). Activation of Galphaq in Cardiomyocytes Increases Vps34 Activity and Stimulates Autophagy. *J. Cardiovasc. Pharmacol.* 69 198–211. 10.1097/FJC.0000000000000461 28376509 PMC5382807

[B23] Mackeh R. Perdiz D. Lorin S. Codogno P. Pous C. (2013). Autophagy and microtubules - new story, old players. *J. Cell Sci.* 126 1071–1080. 10.1242/jcs.115626 23620510

[B24] Maloyan A. Sayegh J. Osinska H. Chua B. H. Robbins J. (2010). Manipulation of death pathways in desmin-related cardiomyopathy. *Circ. Res.* 106 1524–1532. 10.1161/CIRCRESAHA.109.212639 20360253 PMC2890082

[B25] Mearini G. Schlossarek S. Willis M. S. Carrier L. (2008). The ubiquitin-proteasome system in cardiac dysfunction. *Biochim. Biophys. Acta* 1782 749–763. 10.1016/j.bbadis.2008.06.009 18634872

[B26] Mizushima N. Levine B. Cuervo A. M. Klionsky D. J. (2008). Autophagy fights disease through cellular self-digestion. *Nature* 451 1069–1075. 10.1038/nature06639 18305538 PMC2670399

[B27] Pan B. Li J. Parajuli N. Tian Z. W. Wu P. L. Lewno M. T. (2020). The Calcineurin-TFEB-p62 Pathway Mediates the Activation of Cardiac Macroautophagy by Proteasomal Malfunction. *Circ. Res.* 127 502–518. 10.1161/Circresaha.119.316007 32366200 PMC7416491

[B28] Pandey U. B. Nie Z. P. Batlevi Y. Mccray B. A. Ritson G. P. Nedelsky N. B. (2007). HDAC6 rescues neurodegeneration and provides an essential link between autophagy and the UPS. *Nature* 447 859–863. 10.1038/nature05853 17568747

[B29] Pankiv S. Alemu E. A. Brech A. Bruun J. A. Lamark T. Overvatn A. (2010). FYCO1 is a Rab7 effector that binds to LC3 and PI3P to mediate microtubule plus end-directed vesicle transport. *J. Cell Biol.* 188 253–269. 10.1083/jcb.200907015 20100911 PMC2812517

[B30] Pankiv S. Clausen T. H. Lamark T. Brech A. Bruun J. A. Outzen H. (2007). p62/SQSTM1 binds directly to Atg8/LC3 to facilitate degradation of ubiquitinated protein aggregates by autophagy. *J. Biol. Chem.* 282 24131–24145. 10.1074/jbc.M702824200 17580304

[B31] Pattingre S. Tassa A. Qu X. Garuti R. Liang X. H. Mizushima N. (2005). Bcl-2 antiapoptotic proteins inhibit Beclin 1-dependent autophagy. *Cell* 122 927–939. 10.1016/j.cell.2005.07.002 16179260

[B32] Pattison J. S. Osinska H. Robbins J. (2011). Atg7 induces basal autophagy and rescues autophagic deficiency in CryABR120G cardiomyocytes. *Circ. Res.* 109 151–160. 10.1161/CIRCRESAHA.110.237339 21617129 PMC3150753

[B33] Predmore J. M. Wang P. Davis F. Bartolone S. Westfall M. V. Dyke D. B. (2010). Ubiquitin proteasome dysfunction in human hypertrophic and dilated cardiomyopathies. *Circulation* 121 997–1004. 10.1161/CIRCULATIONAHA.109.904557 20159828 PMC2857348

[B34] Ramos F. J. Chen S. C. Garelick M. G. Dai D. F. Liao C. Y. Schreiber K. H. (2012). Rapamycin reverses elevated mTORC1 signaling in lamin A/C-deficient mice, rescues cardiac and skeletal muscle function, and extends survival. *Sci. Transl. Med.* 4:144ra103. 10.1126/scitranslmed.3003802 22837538 PMC3613228

[B35] Rock K. L. Gramm C. Rothstein L. Clark K. Stein R. Dick L. (1994). Inhibitors of the proteasome block the degradation of most cell proteins and the generation of peptides presented on MHC class I molecules. *Cell* 78 761–771. 10.1016/s0092-8674(94)90462-6 8087844

[B36] Rothermel B. A. Hill J. A. (2008). Autophagy in load-induced heart disease. *Circ. Res.* 103 1363–1369. 10.1161/CIRCRESAHA.108.186551 19059838 PMC2607044

[B37] Sano M. Wang S. C. Shirai M. Scaglia F. Xie M. Sakai S. (2004). Activation of cardiac Cdk9 represses PGC-1 and confers a predisposition to heart failure. *EMBO J.* 23 3559–3569. 10.1038/sj.emboj.7600351 15297879 PMC516624

[B38] Schlossarek S. Carrier L. (2011). The ubiquitin-proteasome system in cardiomyopathies. *Curr. Opin. Cardiol.* 26 190–195. 10.1097/HCO.0b013e32834598fe 21415726

[B39] Schlossarek S. Englmann D. R. Sultan K. R. Sauer M. Eschenhagen T. Carrier L. (2012). Defective proteolytic systems in Mybpc3-targeted mice with cardiac hypertrophy. *Basic Res. Cardiol.* 107 1–13. 10.1007/s00395-011-0235-3 22189562

[B40] Singh S. R. Meyer-Jens M. Alizoti E. Bacon W. C. Davis G. Osinska H. (2020). A high-throughput screening identifies ZNF418 as a novel regulator of the ubiquitin-proteasome system and autophagy-lysosomal pathway. *Autophagy* 27 1–16. 10.1080/15548627.2020.1856493 33249983 PMC8526018

[B41] Singh S. R. Zech A. T. L. Geertz B. Reischmann-Dusener S. Osinska H. Prondzynski M. (2017). Activation of Autophagy Ameliorates Cardiomyopathy in Mybpc3-Targeted Knockin Mice. *Circ. Heart Fail.* 10:e004140. 10.1161/CIRCHEARTFAILURE.117.004140 29021349 PMC5679453

[B42] Su H. Wang X. (2010). The ubiquitin-proteasome system in cardiac proteinopathy: a quality control perspective. *Cardiovasc. Res.* 85 253–262. 10.1093/cvr/cvp287 19696071 PMC2797449

[B43] Thottakara T. Friedrich F. W. Reischmann S. Braumann S. Schlossarek S. Kramer E. (2015). The E3 ubiquitin ligase Asb2beta is downregulated in a mouse model of hypertrophic cardiomyopathy and targets desmin for proteasomal degradation. *J. Mol. Cell Cardiol.* 87 214–224. 10.1016/j.yjmcc.2015.08.020 26343497

[B44] Tsukamoto O. Minamino T. Okada K. Shintani Y. Takashima S. Kato H. (2006). Depression of proteasome activities during the progression of cardiac dysfunction in pressure-overloaded heart of mice. *Biochem. Biophys. Res. Commun.* 340 1125–1133. 10.1016/j.bbrc.2005.12.120 16403436

[B45] Wang X. Su H. Ranek M. J. (2008). Protein quality control and degradation in cardiomyocytes. *J. Mol. Cell Cardiol.* 45 11–27. 10.1016/j.yjmcc.2008.03.025 18495153 PMC2574642

[B46] Wauson E. M. Dbouk H. A. Ghosh A. B. Cobb M. H. (2014). G protein-coupled receptors and the regulation of autophagy. *Trends Endocrinol. Metab.* 25 274–282. 10.1016/j.tem.2014.03.006 24751357 PMC4082244

[B47] Weekes J. Morrison K. Mullen A. Wait R. Barton P. Dunn M. J. (2003). Hyperubiquitination of proteins in dilated cardiomyopathy. *Proteomics* 3 208–216. 10.1002/pmic.200390029 12601813

[B48] Zech A. T. L. Singh S. R. Schlossarek S. Carrier L. (2020). Autophagy in cardiomyopathies. *Biochim. Biophys. Acta Mol. Cell Res.* 1867:118432. 10.1016/j.bbamcr.2019.01.013 30831130

[B49] Zheng Q. Wang X. (2010). Autophagy and the ubiquitin-proteasome system in cardiac dysfunction. *Panminerva Med.* 52 9–25. 20228723 PMC2840262

[B50] Zhu H. Tannous P. Johnstone J. L. Kong Y. Shelton J. M. Richardson J. A. (2007). Cardiac autophagy is a maladaptive response to hemodynamic stress. *J. Clin. Invest.* 117 1782–1793. 10.1172/JCI27523 17607355 PMC1890995

