# Supplementary material for: A Transgenic Mouse Model of Eccentric Left Ventricular Hypertrophy With Preserved Ejection Fraction Exhibits Alterations in the Autophagy-Lysosomal Pathway
Source: Front Physiol. 2021 Apr 22;12:614878. doi: 10.3389/fphys.2021.614878 (PMC8121148; doi:10.3389/fphys.2021.614878)
Supplement: Supplementary file 1 [file Table_1.DOCX]

**Supplementary Methods**

**Immunstaining of cardiac sections**

Six micrometer tissue sections (n=3/group) were immunostained using an antibody against mono- and polyubiquitinated conjugates (monoclonal antibody, ENZO, Ann Arbor, USA) followed by AlexaFluor 488 fluorescent secondary antibodies (Invitrogen, Darmstadt, Germany). Slides were also labeled with Phalloidin AlexaFluor 546 (Invitrogen, Karlsruhe, Germany) for cytoskeleton staining and 4′,6-diamidino-2-phenylindole (Roth, Karlsruhe, Germany) for nuclear staining. For quantification of total intensity and number of ubiquitinated aggregates images were taken using the Biorevo BZ-9000 microscope (Keyence, Osaka, Japan) and BZ-X analyzer software (Keyence). After automated capture of 40x magnified images the green fluorescence of protein aggregates was quantified by using the single extraction mode software (BZ-X analyzer software, Keyence), which calculated the results as percent of fluorescence intensity of ubiquitinated proteins related to the entity of the tissue section. The number of protein aggregates per section was analyzed using ImageJ (U. S. National Institutes of Health).
