# Supplementary material for: A Transgenic Mouse Model of Eccentric Left Ventricular Hypertrophy With Preserved Ejection Fraction Exhibits Alterations in the Autophagy-Lysosomal Pathway
Source: Front Physiol. 2021 Apr 22;12:614878. doi: 10.3389/fphys.2021.614878 (PMC8121148; doi:10.3389/fphys.2021.614878)
Supplement: Supplementary file 2 [file Presentation_1.pptx]

## Slide 1
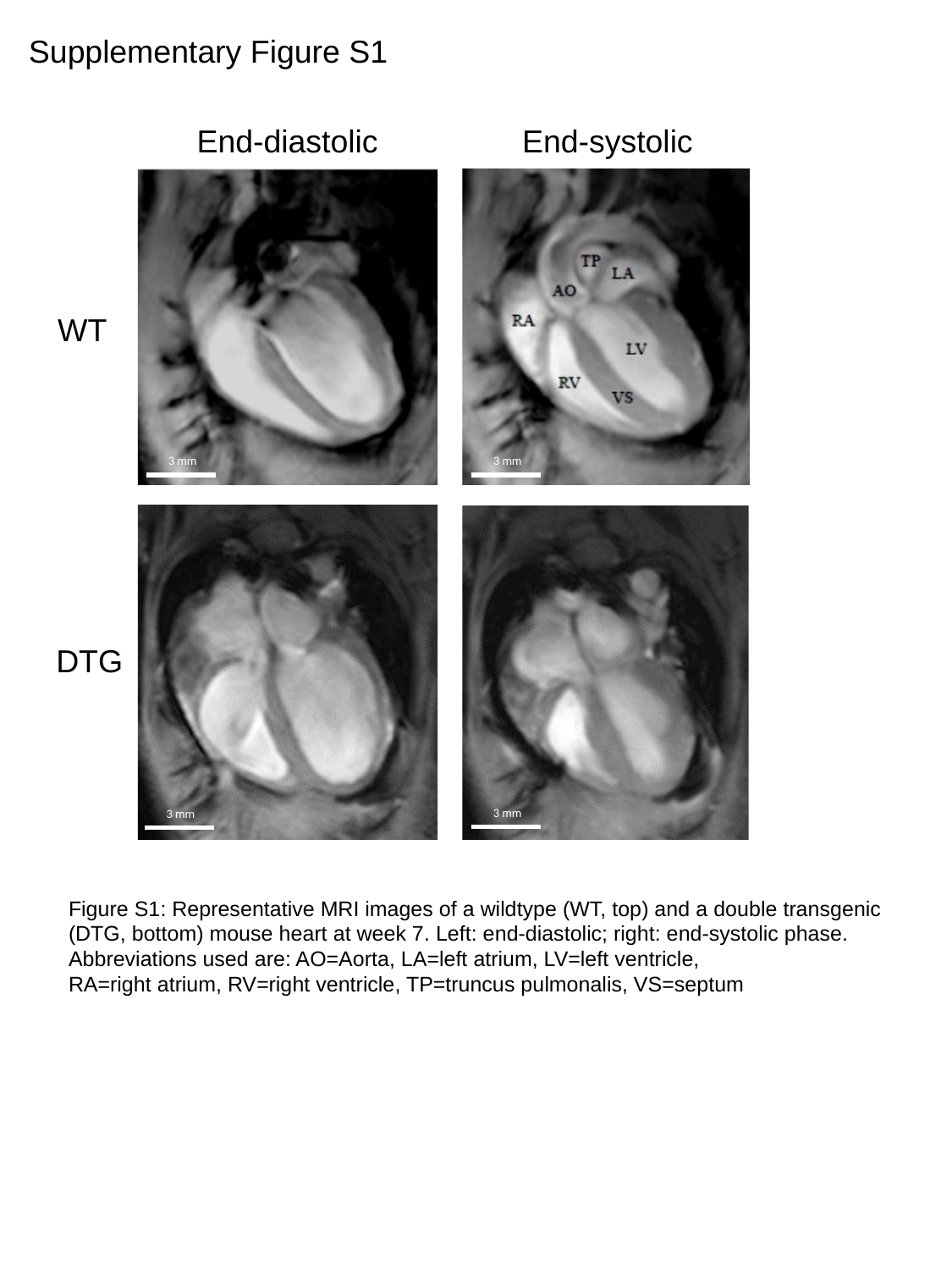

Supplementary Figure S1
End-diastolic
End-systolic
WT
3 mm
3 mm
DTG
3 mm
3 mm
Figure S1: Representative MRI images of a wildtype (WT, top) and a double transgenic (DTG, bottom) mouse heart at week 7. Left: end-diastolic; right: end-systolic phase.
Abbreviations used are: AO=Aorta, LA=left atrium, LV=left ventricle,
RA=right atrium, RV=right ventricle, TP=truncus pulmonalis, VS=septum

## Slide 2
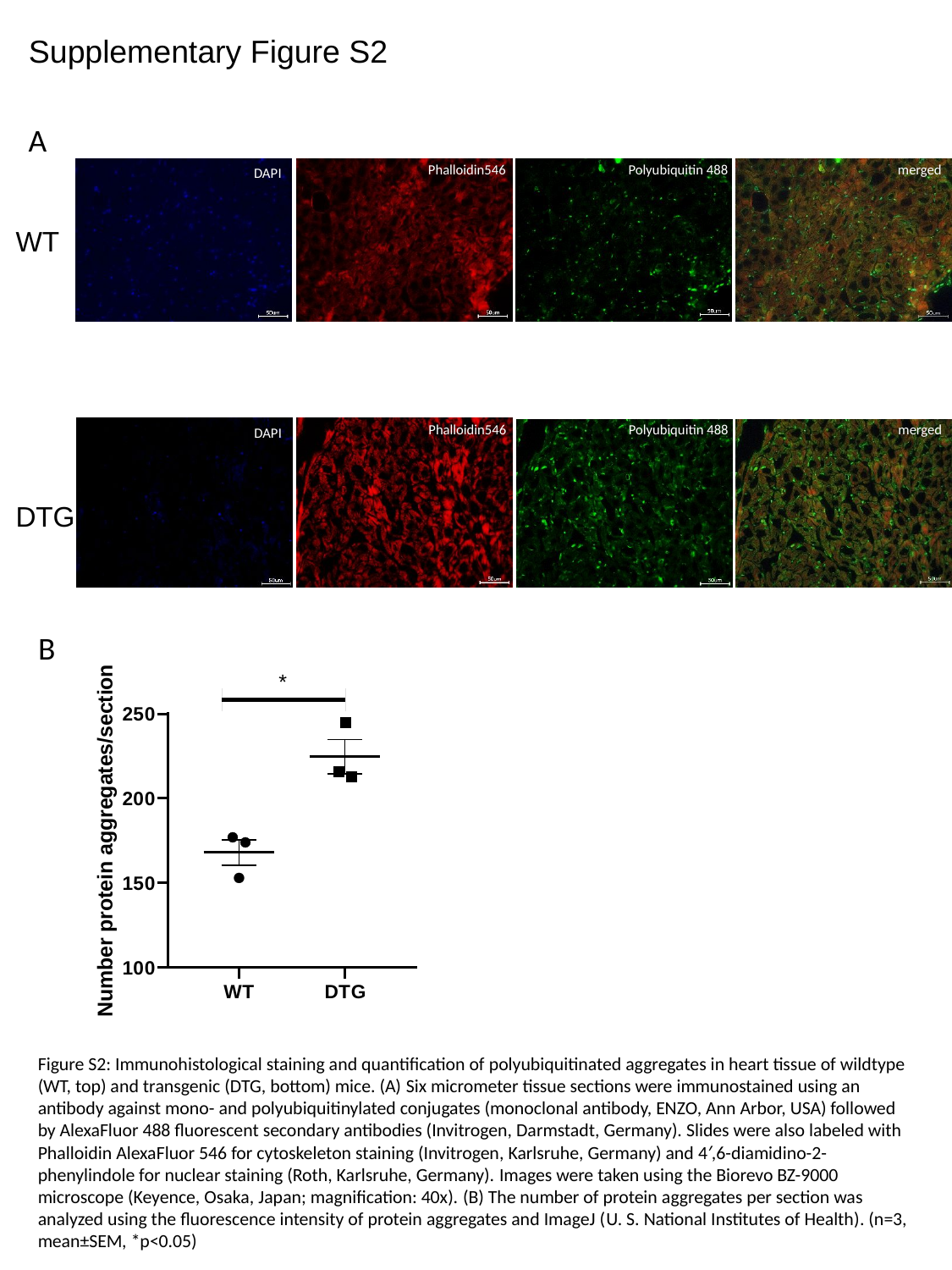

Supplementary Figure S2
A
Phalloidin546
Polyubiquitin 488
merged
DAPI
WT
Phalloidin546
Polyubiquitin 488
merged
DAPI
DTG
B
Figure S2: Immunohistological staining and quantification of polyubiquitinated aggregates in heart tissue of wildtype (WT, top) and transgenic (DTG, bottom) mice. (A) Six micrometer tissue sections were immunostained using an antibody against mono- and polyubiquitinylated conjugates (monoclonal antibody, ENZO, Ann Arbor, USA) followed by AlexaFluor 488 fluorescent secondary antibodies (Invitrogen, Darmstadt, Germany). Slides were also labeled with Phalloidin AlexaFluor 546 for cytoskeleton staining (Invitrogen, Karlsruhe, Germany) and 4′,6-diamidino-2-phenylindole for nuclear staining (Roth, Karlsruhe, Germany). Images were taken using the Biorevo BZ-9000 microscope (Keyence, Osaka, Japan; magnification: 40x). (B) The number of protein aggregates per section was analyzed using the fluorescence intensity of protein aggregates and ImageJ (U. S. National Institutes of Health). (n=3, mean±SEM, *p<0.05)
